# Factors promoting and impeding efforts to deprescribe antidepressants among nursing home residents with dementia– a process evaluation guided by normalization process theory

**DOI:** 10.1186/s12912-024-01932-x

**Published:** 2024-04-28

**Authors:** Sinead Shahrzad, Gritt Overbeck, Anne Holm, Kirsten Høj, Pernille Hølmkjaer

**Affiliations:** 1 Department of Public Health, University of Copenhagen, Section of General Practice and Research Unit for General Practice, Copenhagen, Denmark; 2 Research Unit for General Practice, Aarhus, Denmark

**Keywords:** Process evaluation, General practice, Normalization process theory, Nursing home staff, Dementia, Training, Deprescribing, Antidepressants

## Abstract

**Background:**

Despite recommendations against psychotropic medication in older nursing homes residents with behavioral and psychological symptoms of dementia (BPSD), antidepressants and other psychotropic drugs are still prescribed. We performed a cluster-randomized controlled trial to evaluate the effect of a complex intervention aiming to promote the deprescribing of antidepressants in institutionalized older persons with dementia. To understand the underlying mechanisms of trial outcomes, we conducted a process evaluation exploring the interventions implementation, areas of impact, and contextual factors. The aim of this study was to explore the implementation process and the key factors that promoted and inhibited intervention implementation in the care home setting (Clinicaltrials.gov: NCT04985305. Registered 30 July 2021).

**Methods:**

Qualitative interviews were conducted between August 2022 and February 2023 with four general practitioners and eight nursing home staff from four associated nursing homes in the Capital Region of Denmark. We coded the interview data according to the four constructs of the Normalization Process Theory (coherence, cognitive participation, collective action, and reflexive monitoring).

**Results:**

There was a common understanding of the intervention aim. We observed a raised awareness concerning the deprescription of antidepressants among healthcare professionals with good collaboration (coherence). An overall buy-in to a deprescribing mentality was seen (cognitive participation). There were barriers to the GPs and nursing home staff’s use of the intervention elements and how they implemented it, but to some, a common language was created (collective action). Professionals overall valued the idea of deprescribing, but lack of time, high staff turnover, and low education level among nursing home staff hampered the integration (reflexive monitoring).

**Conclusion:**

Successful implementation seemed to be dependent on the quality of the relationship between the single GP and the single nursing home professional. A common deprescribing mentality promoted the uptake of the intervention. However, several barriers related to lack of resources hindered implementation. It is imperative to adapt complex interventions to the available resources and context.

**Supplementary Information:**

The online version contains supplementary material available at 10.1186/s12912-024-01932-x.

## Background

Dementia results in loss of cognitive functions and primarily affects those above 65 years of age [1]. With the progression of the disease, some may need to relocate to a nursing home [2, 3]. Besides the loss of cognitive functions, up to 90% of people living with dementia experience one or more behavioral and psychological symptoms of dementia (BPSD) [4, 5]. BPSD affects both the person with dementia and caregivers, family, and nursing home staff, causing many healthcare-related issues including challenges with medication, and a higher workload for caregivers and staff [6, 7]. Currently non-pharmacological treatment is the golden standard for BPSD treatment [8–10]. However, many continue to receive multiple medications, including a high amount of psychotropic medication, which includes antidepressants [11–14]. Antidepressants are often used for people with dementia for multiple reasons, but it is uncertain how effective they are [15, 16].

For other psychotropic medication, they also have limited benefits and various adverse events [17–19]. Although it is, to some extent, viable to deprescribe psychotropic medication, multiple studies have only shown varying effects and few concerned antidepressants [20–25].

Various factors may explain the lack of effect and what influences the clinicians in their decision to continue prescribing antipsychotic and antidepressant medication despite the recommendation against this in the guidelines [26, 27]. A factor to consider is communication between general practitioners (GPs) and nursing home staff. In Denmark, in 2016, nursing home GPs were introduced. Since then, around 70% of Danish nursing homes have had an affiliated GP that consults most of the residents [28]. At a nursing home, often multiple and interchangeable nursing home staff are involved in residents’ care and medication which requires clear and straightforward communication. The communication between the GP and staff may result in annoyance and misunderstandings if there is a lack of trust or uncertainties about the appropriate way of caring for the resident [26]. Additionally, concerns among relatives and staff about medication reduction and GPs’ perceived lack of competence in adjusting medication for this patient group are also seen to influence their decisions [26, 29–31]. Some of these challenges are specific to antipsychotic medication and are very difficult to overcome, while others apply to all medication groups. The difficulties that arise from antidepressants may be a bit less challenging to deal with [32].

To address these factors, we developed a complex intervention aiming to reduce antidepressants for nursing home residents with dementia by improving communication and collaboration between GPs, nursing home staff, relatives, and patients [32, 33]. The results of the randomized controlled trial (RCT) will not be able to explain why or why not a possible effect was achieved. A process evaluation addresses the specific context or mechanisms of the outcome and is broadly accepted as an essential part of trials to minimize research waste [33–36].

### cRCT to reduce antidepressants for nursing home residents with dementia

A detailed description of the main trial and the development of the intervention is reported elsewhere [32, 37]. To summarize, the trial was conducted as a cluster randomized controlled trial with GPs working as nursing home physicians as the clusters. In total 21 practices were recruited. Each practice had 1–3 GPs working as nursing home physicians and 1–2 nursing homes with possible patients to include. The practices each included between 3 to15 patients. GPs were randomized on a ratio of 1:1 to intervention and control group by a computer algorithm. Randomization was done after baseline data collection.

Guided by the MRC framework for developing complex interventions, the intervention was developed and tailored in a 5 step process including (1) a literature search, (2) interviews with stakeholders, (3) drafting the intervention prototype, (4) professionals’ assessment of the intervention, and (5) refinement of the intervention [33, 38].

**Fig. 1:**
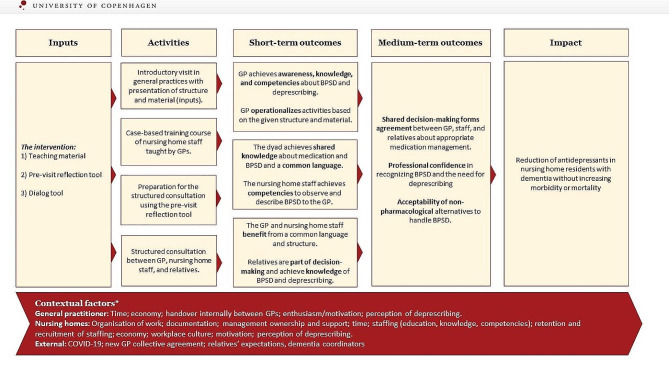
Program theory. (*Contextual factors were identified during the process evaluation)

The intervention consisted of three parts with the overall rationale behind, that it could improve communication and thereby reduce the use of antidepressants (see Fig. 1). The intervention was introduced to the GPs in the intervention group by the research team at individual meetings at the GP practice lasting approximately one hour. The same person from the research team did all the introductions to ensure consistency. Prior to the introduction of the intervention, all GPs (intervention and control) participated in an introductory meeting where experts taught about BPDS, non-pharmacological interventions, and psychotropic medication. This was done to enable GPs in the intervention group to be prepared for the teaching session. The research team did not have any direct contact with the nursing home during implementation.

The three components included:

1. *Teaching material*: A case-based teaching material for the GPs to use at the nursing home in a session with the nursing home staff. The goal of this component was to ensure a common ground between GPs and nursing home staff concerning their knowledge of BPSD and medication. The material included a description of BPSD symptoms, the mechanism, effect, and adverse effects of antidepressants, and a rationale for deprescribing as well as non-pharmacological treatments of BPSD. The teaching material could be adapted by the GPs to suit their preferred teaching style and differ from GP to GP. The teaching session should be held shortly after the trial was initiated, and before any structured consultations were held.
2. *Pre-visit reflection tool*: A checklist and an email template were given to ensure, that; (a) The home visit was planned with members of the nursing home staff who knew the patient best; (b) The nursing home staff were instructed to, contact the relevant relatives and inform them about the home visit if deemed relevant. By using this tool, the nursing home staff had a way to prepare for the structured meeting and share those thoughts with their colleagues to obtain a more nuanced picture of the patient and the nursing home staff’s view of the patient. The pre-visit reflection tool was introduced by the GP to the nursing home staff either at the teaching session or at one of the regular visits from the GP at the nursing home. In collaboration with the GP, the nursing home staff decided how to best report back with the results (electronically or in paper form).
3. *Dialog tool*: The dialog tool included a list of questions to help the GP explore the nursing home staff’s and, if relevant the patients’ and the relatives’, concerns and views on deprescribing antidepressants. The goal of the tool was to aid the GPs in obtaining a structure for a consultation with a focus on antidepressants and to ensure that the views of nursing home staff and patients/relatives were included. Furthermore, it included questions to prompt communication concerning when and what to report back to the GP, to ensure proper follow-up. The tool was only introduced to the GP by the research member at the introductory meeting and the GPs were encouraged to use it and adapt it to their own style if relevant.

The intervention was conducted between October 2021 and December 2022 in the Capital Region of Denmark. The purpose of the intervention was to reduce the number of antidepressants for institutionalized persons with dementia through an improvement of communication and collaboration between GPs, nursing home staff, relatives, and patients. The primary outcome was any reduction of antidepressants. Secondary outcome measures included differences in the use of other psychotropic medication, mortality, morbidity, and severity of BPSD.

The aim of the process evaluation was to explore the key factors that promoted and inhibited the implementation of the intervention from the viewpoint of the GPs and nursing home staff.

## Methods

### Study design

We carried out the data collection for the process evaluation in parallel with and shortly after the finalization of the RCT. The overall aims of this study were to explore intervention usability and implementation processes, including key factors that promoted and inhibited intervention implementation. We used the NPT as the theoretical framework throughout the study as a means to frame and evaluate the findings.

Based on qualitative data on participants’ experience of the intervention, the Normalization Process Theory (NPT) was used to identify key facilitators and barriers that promoted or inhibited the core elements of the intervention [39, 40]. NPT is a middle-range sociological theory commonly used in Implementation Science to understand how and why new interventions are implemented (or not implemented) in organizational practice by drawing attention to four analytical domains: making sense of the intervention (coherence), engagement in the intervention (cognitive participation), work done in the intervention (collective action), and individual adaptation of the intervention (reflexive monitoring) [40]. Where MRC was primarily used in the planning of the process evaluation, the NPT was used from the planning phase and actively throughout the study as a means to frame and evaluate the findings.

This study has been reported in accordance with the consolidated criteria for reporting qualitative research (COREQ) (Additional material 1) [41]. The main cluster randomized trial including the process evaluation was submitted to the Research Ethics Committee of the Capital Region of Denmark. As defined by the Danish Act on Research Ethics of Research Projects Sect. 2, the project does not constitute a health research project but is considered a quality development project. Therefore, both studies do not require approval from the Committees on Health Research Ethics of the Capital Region of Denmark (Journal no: H-20,084,023). Informed consent was obtained verbally from all participants before the study procedures. The participants were informed about all study proceedings pertaining to the process evaluation verbally and in writing. Written information about the main cluster randomized controlled trial was given to the participants when they enrolled.

### Setting and participants

The process evaluation took place in the Capital Region of Denmark. All GPs and their respective nursing homes participating in the intervention arm of the RCT were eligible for the process evaluation. All GPs were nursing home GPs. The nursing home staff included nurses, healthcare assistants, healthcare helpers, and substitute staff. Healthcare helpers have a 20-month introduction followed by 15 months of specific education after their compulsory education. Healthcare assistants also have a 20-month introduction, but their specific education lasts 34 months after their compulsory education. Nursing home staff was only invited to participate if the GP had agreed to participate. The GPs could apply for reimbursement for the length of the interview, but no reimbursement was available for the nursing home staff, as the interviews were performed in their working hours.

Of the eleven GPs in the intervention group, three GPs had never initiated the intervention, leaving eight who were still active in the study at the time of conducting the process evaluation. These eight GPs and their accompanying nursing home were invited via email to participate. During this stage, two additional GPs withdrew from the main study. One of the clinics had never initiated the intervention. The other clinic was excluded since they had no eligible patients since a psychiatrist worked permanently at the nursing home and our inclusion criteria stated that patients were excluded when consulting a psychiatrist. This left six remaining GPs to take part in this process evaluation. Of these six, two did not respond despite several attempts at communication. When a GP responded positively to the request to participate, the associated nursing home was invited via email and followed up through a phone call. In this process evaluation, mostly nurses and healthcare assistants were present for interviews. In total, we included 12 participants, including four GPs and eight staff members. GPs were interviewed individually and the nursing home staff mostly in groups. Deviations from the protocol were made, since we originally planned to conduct interviews during the implementation period including observation from the teaching sessions and to include 10 GPs, 10 nursing home staff and 10 relatives. The deviations were due to different reasons, which are further elaborated in the discussion sections.

### Data collection

Two topic guides based on NPT’s constructs were developed (SS and GO) and semi-structured, qualitative interviews were performed. The two topic guides were similar in topics concerning how they perceived the intervention but differed according to the part of the intervention that was specifically used by either the GP or the nursing home staff. The topic guide used for the GPs also included questions regarding the reasons for their participation in the study, the startup session in which the nursing home staff did not participate, and how they experienced using the dialog tool. The nursing home staff had questions regarding how they perceived the teaching sessions from the GP and the use of the pre-visit reflection tool. The topic guides were developed based on the four NPT domains, with sub-questions being guided towards their use of the individual intervention elements and overall experience (Additional material2). The interview guides were not pilot-tested due to logistical reasons.

All interviews were conducted by the first author SS (Medical Anthropologist with special knowledge of health technology and both training and practical experience with interview techniques) and took place in the GP offices and at the nursing homes. No one besides the participants and interviewer was present. In a single case, a nursing home staff member was interviewed over the phone to best accommodate their availability. Researcher SS had no prior relationship with the GPs or nursing homes.

Participation in the interviews was voluntary and participants were only interviewed once. For each interview we continued until no new information was shared. The interviews were audio recorded and transcribed verbatim by SS. No field notes were taken. The recordings were not shared with anyone outside the research team.

### Data analysis

The transcribed data was uploaded into NVivo (release 1.6.1) in Danish for data management and analysis. The analysis was guided by the aim of the process evaluation and set within the NPT framework, using the framework for the analysis of the interview. We began analyzing data after conducting the first few interviews, continuing this analysis concurrently with subsequent interviews. The first author SS coded the data from both GPs and nursing home staff and analyzed first line-by-line using inductive coding to identify factors influencing the implementation including barriers and facilitators set within the NPT domains. Random samples were checked by the other authors PH and GO to obtain valid coding and all codes were discussed in the author group. The coding tree was designed based on NPT and visualized in Additional Material 3. The data was initially separated into a GP and nursing home staff ‘super-category’, which each held the four components of NPT. Subcategories were then created to encompass their individual ‘coherence’ and ‘cognitive participation’ of the intervention elements.

During the coding process, it did, however, become unnecessary to use the subcategories branching from the GPs ‘cognitive participation’ strand, since it was not expressed in the interviews (crossed over in Additional Material 3). Anonymized, translated quotes are used illustratively. We did not conduct member checking as part of our verification process [42]. The quotes were translated by SS in collaboration with GO. They had no distinct formal training in translation, but GO has vast experience with conducting interview studies and translating quotes from Danish to English.

## Results

**Fig. 2:**
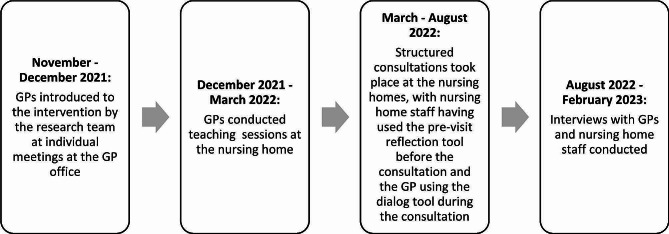
Timeline of the intervention’s implementation for the GPs and nursing home staff that participated in the process evaluation

First, interviews with GPs took place, since the intervention was delivered to them and they had to initiate the implementation in collaboration with the nursing homes. These interviews were conducted between August and December 2022. During this time, the main trial was still running, but no additional structured consultation could take place. Because of difficulties in setting up meetings with the nursing home staff, all their interviews were conducted in February 2023 after the trial was completed. Figure 2 describes the overall implementation of the three parts of the intervention and the timeline for data collection of the process evaluation. The time range of the GP interviews was 20–35 min with a mean duration of 27:30 min. The time range of the nursing home staff interviews was 18–27 min with a mean duration of 22 min. Throughout this paper, we will be addressing the participants by either individual GP (GP 1–4), by individual nursing home (NH 1–4), or by the collective dyad of GP and accompanying nursing home staff (dyad 1–4). Table 1 describes the main characteristics.

**Table 1:** *Characteristics of the participating dyads*

Dyad	General practice	Years of experience as a GP	Age	Total number of residents at the nursing home	Number of patients that the GP attends to at the nursing home	Number of patients included in the RCT*	Nursing home	Qualifications	Age
1	GP 1.	24	64	55	55	8	NH 1. 3 participants NH 1.1, NH 1.2, NH 1.3	Assistant nursing home manager Healthcare assistant Nurse	50 37 54
2	GP 2.	6	43	48	35	7	NH 2. 2 participants NH 2.1, NH 2.2	Healthcare assistant Healthcare assistant	43 45
3	GP 3.	16	50	85	30	5	NH 3. 1 participant NH 3.1	Nurse	*Mis-sing*
4	GP 4.	19	57	98	35	12	NH 4. 2 participants NH 4.1, NH 4.2	Healthcare assistant Nurse	49 53

GP: General Practitioner, NH: Nursing home. *Only patients with dementia (diagnosed or suspected), taking antidepressants, not terminally ill, or receiving care from a psychiatrist could be included

**Fig. 3:**
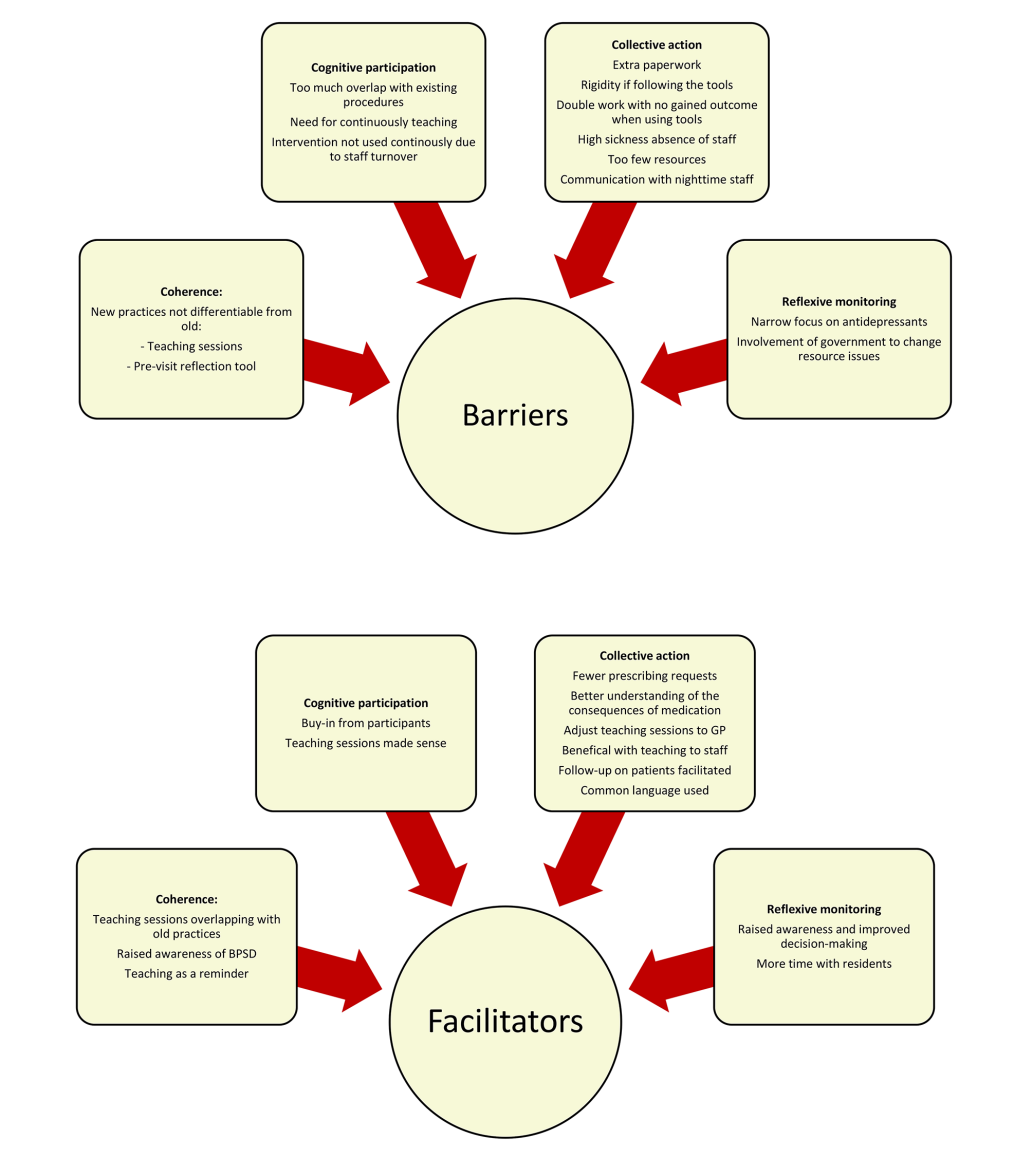
Summary of results within the NPT-domains

The analysis identified several factors influencing the implementation process. Overall, these factors can be divided into barriers and facilitators each within a domain of the NPT as elaborated on below. The results are presented according to the NPT domains and when relevant, sub-constructs (presented in *italic*), while acknowledging that there is an overlap between the domains [43]. Additionally, some results may present both a facilitator and a barrier depending on the context. The main findings are presented in Fig. 3 with barriers and facilitators according to the four NPT domains.

### Coherence: how participants made sense of the intervention

Barriers related to coherence involved how professionals *differentiated* the new practices from the old practices. The GPs were unsure about the details of the intervention introduced without being reminded of the teaching materials, pre-reflection tool, and dialog tool by the interviewer. Some nursing home staff members had trouble pinpointing the individual uses of the intervention elements. When addressing the teaching sessions they were acknowledged as a new practice but with some overlap with old practices already in use by the nursing home staff and the GP which was seen as both a barrier and a facilitator.

A facilitator within *communal specification* was that both GPs and nursing home staff had a raised awareness of symptoms related to BPSD which also served to create a common understanding of the intervention.

Another facilitator from the sub-construct of *individual specification* was the overall understanding of the GPs in relation to the teaching session, which they saw as useful.

In some nursing homes, the *internalization* of the pre-visit reflection tool was seen as a barrier. The nursing home staff experienced that when they already had a similar tool, they did not use or identify the intervention as something useful.

*“The Municipality… has worked with the BPSD model for a long time, I have had one of these forms [pre-reflection tool] before, and I simply just sent that one to [GP 1], because there is no reason to do the same work twice.” (NH 1.1)*.

*“But we have always done that? That is not something we are doing because of this project. It is nothing new. (NH 2.1)*

However, using teaching as a reminder could also reinforce the pre-existing mentality and act as a facilitator

*“I think that there has been a lot of focus on it actually working [person-centered approach], […] But it is always nice to revisit things.” (NH 4.2)*.

Another facilitator was the overlap between the person-centered approach to dementia care that was already used and the alternative treatments taught by this project in the teaching sessions could complement each other

“*When we work with Tom Kitwood daily, it becomes really easy [to adopt the new methods], because we are trained in that approach.”* (*NH 4.1*).

### Cognitive participation: how participants engaged and committed to the intervention

Concerning *enrollment*, s*ome* GPs and nursing home staff felt that parts of the intervention were not relevant to reorganizing their current workflow around, since there was too much overlap, and therefore viewed as a barrier. This concerned the pre-reflective tool and dialog tool, which covered procedures that already existed at the nursing home and therefore hindered the commitment to the intervention

*“I think that it’s just… It’s just a part of us, we just do it when we encounter something new. And then we advise the paperwork and have a dialogue with the GPs.” (NH 2.2)*.

Despite these barriers, acting as a facilitator was the buy-in to the intervention and change of procedure which was welcomed by GP/nursing home dyads.

A facilitator for the implementation was the *legitimization* encouraged by both GPs and nursing home staff about the teaching material. They were positive and receptive to the teaching sessions and saw the reason for the material and the education that followed with it.

*”There were many [nursing home staff] who were really positively surprised about learning what medicine actually does to a dementia brain and stuff like that… So, I think it [the teaching sessions] really made a lot of us move our focus from medicine onto other alternatives.” (NH 4.2)*.

*“They [nursing home staff] were also interested in joining the project. So, what happened was, I taught about the same things that I had been taught, and then we had a discussion… The thing about being a little more careful and not blindly trusting the medicine, right? (GP4)*

Another barrier was concerning the *activation* where several nursing home staff expressed a wish to have had more and continuous education on the BPSD to account for the changing staff. Furthermore, it was difficult for nursing home staff to effectively use the intervention daily continuously due to staff turnover at the nursing homes.

*“I think it was only at the beginning when we did those reflections on the residents that had to be made changes on, and then I don’t think we have experienced much more. Well, at least I have not. Maybe the first month, when we started it here, I think there was a focus on it.” (NH 1.2)*.

If there had been a dedicated resident coordinator functioning as a champion, it could have kept the engagement.

### Collective action: how the intervention was enacted

Some variation occurred as to how many of the intervention elements were used by the dyads, as seen in Table 2.

Most dyads collaborated in selecting the participants with each part having their expertise. Three of four GPs conducted the teaching session, but only a few dyads actively used the pre-visit reflection tool and just half of the GPs used the dialog tool.

**Table 2:** *How the dyads used the intervention elements*

	Dyad 1		Dyad 2		Dyad 3		Dyad 4	
Intervention elements	GP 1	NH 1	GP 2	NH 2	GP 3	NH 3	GP 4	NH 4
Teaching material	not used	not used	used	used	used	used	used	used
Pre-visit reflection tool	used	used	used	used	not used	not used	not used	not used
Dialog tool	not used	-	used	-	not used	-	used	-

GP: General practitioner, NH: Nursing home

For *interactional workability* the introduction of the pre-visit reflection tool and dialog tool was a barrier to the implementation since it added excess paperwork. Several GPs found the systematic of the tools to be too rigorous.

*“Yes, I think I remember that one, but sometimes it’s difficult to determine how much time you want to spend on the side. Because I think you can get a little too tied down by sitting with those things [dialog tool].” (GP 3)*.

Some nursing home staff had their routines and version of the pre-visit reflection tool, which created double work and no gained outcome. The fact that some nursing homes already had awareness and procedures aimed at alternative dementia treatments made it difficult for the nursing home staff to adjust to the intervention elements.

*“It has not been useful at all, it has made our work a tiny bit more annoying since what we already have, we have not been able to use. Therefore, this project has cost us double the work…. So, it has been staggeringly annoying. Because no better product has come out of it. It is a bit stupid that you do not try to incorporate the systems, which we already have.” (NH 1.1)*.

However, it was facilitated in the GPs’ experience since they received fewer prescribing requests from the nursing home staff. With a better understanding of the consequences of antidepressants in the nursing home staff, they were less prone to ask the GP for medication.

*”Well, we benefitted from properly following up on the patients. Often you make a prescription and then everyone forgets about it. After all, we found out that if we had an agreement to do something, we could more easily talk about it and what had happened. What we’ve achieved is that the intervention makes it easier to follow up with patients that we’ve started to deprescribe.” (GP 2)*.

*“Well, I think they [nursing home staff] have gotten better at not begging for medicine. Like, when a nurse calls and says: now I absolutely think we need to give him some antidepressants! So they were more in tune with my train of thought than driving off on the other side. They are also very interested in doing the right thing.” (GP 4)*.

A facilitator within the context of *relational integration* was that the GPs were able to adjust the teaching sessions to fit their teaching method which made them useful. Furthermore, the sessions were beneficial when there were many unskilled laborers and substitute staff since they did not have the same qualifications as nurses or healthcare assistants.

*“Yes. I actually think, at least, that we know what psychotropic medication does to the brain, but especially the healthcare helpers and uneducated staff have difficulty understanding why we can’t just do this [use psychotropic medication] (GP 4.1)*.

Concerning *Skill set workability* a facilitator expressed by the professionals was that it was easier to periodically follow up with patients who were on a deprescribing trajectory compared to those who were not. The use of a common language that had been established when making collective agreements about a patient’s changes in medicine, facilitated the implementation further on.

A barrier to the *contextual integration* of the intervention was during periods of high sickness absence among the nursing home staff. Even though initial communication worked, frequent turnover of substitute staff made it hard to maintain a work protocol creating an important contextual factor.

“*The problem, which has also been evident while this project has been going on, is a lot of sicknesses occurred among the nurses, therefore we had many temporary staff and substitutes. That is a problem, because many temporary workers [at the nursing home] do not have the same qualifications to give relevant observations of the residents, and this makes for somewhat difficult working conditions.” (GP 3)*.

Further, the intervention depended upon the nursing home management and the work environment. If there was too much turnover in staff and the resources were low, these factors constituted important barriers to enacting a new practice.

*“For us, it is the case that one department of the nursing home runs a little more chaotically and the other one runs very quietly…, if there is a stressful work environment it is difficult to discontinue medication…. So you will probably have to, when you have to do that, also look at what resources are available in the nursing homes. How much can it accommodate?” (GP 2)*.

Another barrier was the issue concerning internal communication between the daytime and nighttime staff at the nursing homes. Many nursing homes operate with a separate staff unit during the night who do not necessarily participate in meeting with the GP and thus do not gain nor share knowledge directly with the GP leading to unequal distribution of work.

### Reflexive monitoring: participants’ reflections on the intervention

In general, most participants experienced having a raised awareness and the ability to make educated decisions as the main outcome of this intervention, however, most dyads had not created new routines or practices.

The deprescribing process enabled the staff to relocate their resources and spend more time with the residents. This made it possible to be present and calming instead of prescribing medicine, although it required some extra work.

*“I mean, it has affected our work on those days when there are really restless people. We try to meet their needs as much as possible by dividing the days up so that the resident who really has this extra caring need gets more one-on-one time with a staff member…Of course, it takes up a little extra when we do it, but it is well spent in the long run.” (NH4.1)*.

A GP addressed the issue of a narrow focus on antidepressants as a problem.

*“I think it can be a very complex issue, because they are also suffering from a lot of other things, right? Therefore, it is hard just to talk about these [antidepressants]. After all, there are many other medicines that you could also take a close look at. In general, I think this consideration that you can probably go in and revise or at least look critically at medicines in this patient group is useful.” (GP 3)*.

For GPs and nursing home staff initiatives from the governmental level were warranted. Since healthcare helpers and substitute staff are the primary providers of basic nursing care and the ones spending the most time with the residents with BPSD, they are also the individuals in need of the most education and intervention elements. However, given the frequent turnover of staff and differing levels of commitment to the nursing homes, on-site education, as tried in this intervention, becomes difficult.

*“But the more skilled staff you have,… the more, you will be able to reduce medication consumption. In other words, when a new face comes along all the time,…the more difficult it is.” (NH 4.2)*.

*“It is, after all, better education of staff and better working conditions. It’s something as simple as, we have residents with dementia, who become volatile, who look for the doors and look out, look away, and can be aggressive when they can’t figure it out. The thing is, it’s about being able to accommodate them and that is mainly solved with pedagogy. Pedagogical training and more resources. It is not on us doctors, no.” (GP 1)*.

## Discussion

Our objective in this process evaluation was to investigate the factors that impede or promote the introduction of a complex intervention to reduce antidepressant prescriptions in nursing homes.

Within the four analytical dimensions of the NPT, several factors promoting and inhibiting the uptake of the intervention were identified. Factors promoting the introduction of the intervention elements included: A raised awareness of a deprescribing mentality and using the teaching material was valuable for especially the temporary or uneducated staff (coherence). It also created an increased buy-in to a systematic approach to detecting where deprescribing is appropriate (cognitive participation) and gave the GPs and nursing home staff a common language as well as prompted more visible follow-up (collective action). A previous good relationship between the GP and nursing home staff was a key factor in successfully working with the new routine.

Though we had performed a tailoring process of our intervention, we identified a number of factors impeding the implementation: a high staff turnover making it difficult use the elements of the intervention in daily work (cognitive participation). A lack of internal coordination at the nursing homes including communication with the GP and clear work division (collective action). Excess paperwork for both the GPs and nursing home staff (collective action). Poor education level among nursing home staff (collective action) and a narrow focus on antidepressant medication including missing initiatives from a governmental level (reflexive monitoring).

The facilitators and barriers also aided in the interpretation of how the intervention has affected the short-term and medium-term outcomes presented in the program theory. According to the short-term outcomes, GPs and nursing home staff should have achieved more awareness and knowledge, and benefitted from a common language concerning BPSD and deprescribing. They achieved it to some degree, as represented by the facilitators under coherence and collective action. However, issues concerning coherence, cognitive participation, and collective action, including teaching and communication, highlighted an incomplete change. The same holds true for the operationalization of the parts of the interventions, which did not always result in structures that helped the process. Furthermore, the relatives were not actively participating, thus making this short-term outcome completely unmet.

According to the varying effects based on the short-term outcomes, it is only possible to conclude that the medium-term outcomes are met to a low degree. In some cases, shared decision-making occurred, but without the involvement of patients or relatives. We observed increased professional confidence for the GPs to a certain degree, but it is uncertain how much it has changed for the nursing home staff.

Overall, the lack of fulfilling some of the outcomes can very well have led to a lesser reduction of antidepressants in the main trial.

This study provides important insight into the behavior of the participants concerning a deprescribing practice for antidepressants. Several other studies have addressed the importance of understanding how something is implemented [44, 45]. In our study, it was evident that nursing homes with good communication and collaboration between GPs and nursing home staff were more likely to use the intervention. Having good communication between professionals has been proven to minimize the risk of making mistakes [46, 47].

Other complex interventions have shown the value of training or teaching to increase the knowledge of the participants and to ensure a common language that aids the uptake of intervention into everyday practice [48, 49]. This was also important within our study and added to the common language and understanding of the new mentality, especially for the uneducated and temporary staff. However, the teaching session was only held in the beginning and since there was a high turnover of staff, some of the value may have been lost. Similar findings concerning high staff turnover within the nursing home field and the need for continuous teaching are seen in the literature [50]. The high staff turnover may also have been a factor in the level of confusion concerning the individual forms and tools that were present within the intervention, which has been shown to hinder interventions in nursing homes [51, 52]. Furthermore, in some nursing homes additional overlapping education on BPSD was conducted as part of a focus area within the nursing home. This education was provided from other sources than what was taught in the intervention and it was not possible to restrict the use. Because of the time delay from receiving the teaching session and the interview with the nursing home staff, some issues with differentiating the different educational sessions occurred, thus limiting the actual understanding of the practice attempted to be implemented in our study.

In those nursing homes where the GPs and nursing home staff managed to implement a part of the intervention that prompted a “new shared language”, the intervention enabled a shift of focus towards deprescribing and why it is important. This improved the communication of medication needs and the workload for the GP who was prescribing medication less frequently. This improvement made the resident’s needs more visible and made it possible for the nursing home staff to make necessary follow-up adjustments, as seen in other studies [53]. Another issue raised by the GP was when a strict focus is on one medication or condition, there will be something else that receives less focus. This is also addressed in studies with medication review, where too many focus areas may result in fewer things being changed [53, 54].

Improving collaborative care might also have benefitted the intervention to ensure that the residents receive the best quality of care [52, 55]. The increase in workload with no added value for the staff is often seen in complex interventions. This is an important factor to consider when implementing a new practice since it can become an influential barrier [47].

In order for a new practice to be implemented, it has been shown to be important with good coordination between all active actors [44]. An active nursing home coordinator or champion responsible for keeping track of the new practice and keeping the practice consistent might have benefitted the intervention. Both for the longevity of the new deprescribing mentality but also to ensure everyone, both night and day staff, are aware of the new practice.

### Strengths and limitations

By evaluating the intervention as we have done in this study, it becomes transparent what mechanisms are important when implementing a deprescribing tool and what needs to be taken into consideration when upscaling. This study also aids in understanding the distribution and magnitude of the issues of implementation. Using NPT as a theoretical framework is a strength in this process evaluation since it provides a theory-informed analysis and interpretation of several facilitators and barriers while revealing the complexities of roles in health care.

A major limitation of the study was the deviations from the protocol which occurred. Originally, we had planned to conduct the interviews during the implementation process, but the interviews were conducted more than a year after the intervention was initiated. This was due to leave of absence and staff turnover in the research group. Consequently, several participants had trouble recalling the project details. Another limitation was that we did not get observations from the teaching session, which limited our insights into what was actually taught, since adaptation was possible. Also, fewer participants than originally planned were included and no relatives were included. Since only 11 GPs were in the intervention group, and 5 had not specifically used the intervention, it was not possible to interview the predefined number, since we wanted to evaluate the implementation process. Although interviews with GPs who had not completed the intervention would have been relevant to understanding the factors that had inhibited them from completing or even beginning the intervention, the latency of the interviews made it difficult to ask them to recollect why they did not use the intervention. Furthermore, neither residents nor relatives were interviewed, which could also have provided important insights. However, since they were not actively part of the intervention in the final form because their mandatory presence was removed in the development process, it was less relevant for the evaluation [32]. As for the patient’s perspective, a recent study concerning nursing home resident’s thoughts on discussing deprescribing has shown that residents may not know how to talk about their medication [56].

Due to logistical reasons, we did not pilot test the topic guide, which is a limitation. A pilot test especially when using the NPT as a framework could have given valuable insight into which areas that may have needed more probing from the participants following the four analytical domains.

Another limitation was concerning data saturation. In our study, we undertook a series of strategic measures to ensure data saturation in our small sample of interviews, aiming for a comprehensive understanding of the research topic.

We documented the process of data collection and analysis thoroughly, including how and when we determined that saturation was reached, ensuring the credibility of our study. Although we started with a small sample, we remained open to increasing the number of interviews if new themes continued to emerge and saturation had not yet been achieved [57]. By adopting these measures, we aimed to enhance the likelihood of achieving data saturation, ensuring that the data collected comprehensively addressed our research questions, even with a relatively small number of interviews. The absence of member checking, however, is acknowledged as a limitation that could potentially impact the depth and validation of our findings.

## Conclusion

Process evaluations are a vital part of understanding complex interventions. In this study, we uncovered a relatively low uptake of the intervention by GP/staff dyads and an overall uncertainty about how to collaborate appropriately about the deprescribing of antidepressants. The low uptake of the intervention may have been partly due to less than ideal implementation and follow-up by the research team with the study being anchored in general practice and including nursing homes more. Another major reason was the high staff turnover and low educational level among staff, which makes the introduction of a common language and better training of staff in nursing interventions on BPSD challenging. This study contributed to the uncovering of the mechanisms related to the uptake of the deprescribing intervention.

Overall, more barriers than facilitators were observed. The attempt to encourage the involvement of nursing home staff in the deprescribing process was achieved to some extent, but there were still many reports of GPs and nursing home staff being unsure about their role and participation. This study adds to the knowledge that effective implementation of a deprescribing mentality is dependent on a functioning GP/nursing home dyad with limited staff turnover and proficiently educated staff as well as an implementation strategy involving both GPs and nursing homes. These things are often outside the scope of this type of research and call for a more governmental intervention to improve the resources within the area before further improvements may happen.

### Electronic supplementary material

Below is the link to the electronic supplementary material.

Supplementary Material 1

Supplementary Material 2

Supplementary Material 3

## Data Availability

Data may be made available through formal data sharing agreement with the authors’ institution on reasonable request.

## Abbreviations

BPSD: Behavioral and Psychological Symptoms of Dementia
GP: General Practitioner
MRC: Medical Research Council
NH: Nursing Home
NPT: Normalization Process Theory
RCT: Randomized Controlled Trial
