# Supplementary material for: Factors promoting and impeding efforts to deprescribe antidepressants among nursing home residents with dementia– a process evaluation guided by normalization process theory
Source: BMC Nurs. 2024 Apr 28;23:287. doi: 10.1186/s12912-024-01932-x (PMC11057106; doi:10.1186/s12912-024-01932-x)
Supplement: Supplementary file 1 — Supplementary Material 1 [file 12912_2024_1932_MOESM1_ESM.pdf]

Additional material 2: Topic Guides

TOPIC GUIDE – GP

Brief presentation of the purpose of the interview - because you are the driving forces in the intervention, it is important for me and the research to get you to put into words and elaborate on the process around the intervention. Briefly about me and my role - I have not been involved in developing the intervention, so I am therefore trying to form an overview as an outsider.

There is 1 hour allotted for the interview, but let me know if you have to leave soon, so I can take it into account.

I just want to let you know that I will be recording this interview. Audio files and transcriptions will be destroyed in 2 years, and will of course be stored confidentially and securely according to GDPR legislation. All of your used statements will be made anonymous. Is this something that you want to consent to?

Do you have any questions before we get started?

First of all, I would like to ask you for a brief introduction of yourself

Name and occupation.

[START]

• Why did you agree to be part of the project?

• How did you experience the start of the intervention? You were invited to a training course - what happened then?

• How did you contact your nursing home?

• How did you find patients for the project?

- Was it difficult/easy?

• How would you describe your collaboration (GP/NH) before the intervention?

- (How do they understand "cooperation"?)
- What was difficult/easy about the collaboration?

[UNDERSTANDING/COHERENCE]

• What were you told on the course? What was the idea of the intervention? (October 2021)

• How was the course?

[ENGAGEMENT/COGNITIVE PARTICIPATION]

• What happened during the course of implementing the intervention?

• How did you keep the commitment running? Was a procedure put in place?

Ongoing contact with nursing homes

• Who were you in regular contact with at the nursing home and why?

• Who wanted and who didn't want to invest in the intervention?

• What was it like to engage the nursing home staff?

Everyday practice

• How did it fit into everyday practice?

• Were there others in the clinic, who were involved in the intervention?

Obstacles?

• Why didn't it work?

• Try to put into words the elements that have been an obstacle.

[COLLECTIVE ACTION]

This part is about how useful the material has been. We will talk about the teaching at thenursing homes, your preparation for the visits and your dialogue tool.

Nursing home teaching (of symptoms)

• Did you teach at the nursing home?

• How did your teaching at the nursing home go?

• Who from the nursing homes participated?

• Were they receptive towards it?

• What did you get out of doing the teaching?

• Did the care home staff request more teaching/other teaching?

Pre-visit reflection tool

You received this form.

• How was it put into work?

• How did the well-being scheme work?

• What was used/not used?

• What did you do before visiting the patients? Did sending the email work?

• What did you get out of the reflection?

Dialogue

• How did the dialog tool put into work?

• What was used/not used?

• What did you get out of the dialogue tool?

[ASSESSMENT/REFLEXIVE MONITORING]

• Compared to before the intervention - how has your collaboration (GP/NH) changed?

We assume that you have signed up for this project because you are interested in reducing medication.

• From your chair, what would it take for less psychotropic medication to be prescribed?

• Why doesn't it happen?

• Have you experienced it happen? Elaborate.

• Where do the nursing home and the relatives fit into this? What is required of the collaboration?

• Do you have anything to add about your experience of the intervention here in conclusion that we have not covered?

TOPIC GUIDE – NH Staff

Brief presentation of the purpose of the interview - because you are the driving forces in the intervention, it is important for me and the research to get you to put into words and elaborate on the process around the intervention. Briefly about me and my role - I have not been involved in developing the intervention, so I am therefore trying to form an overview as an outsider.

There is 1 hour allotted for the interview, but let me know if you have to leave soon, so I can take it into account.

I just want to let you know that I will be recording this interview. Audio files and transcriptions will be destroyed in 2 years, and will of course be stored confidentially and securely according to GDPR legislation. All of your used statements will be made anonymous. Is this something that you want to consent to?

Do you have any questions before we get started?

First of all, I would like to ask you for a brief introduction of yourself

Name and occupation.

[START]

• How did you experience the start-up of the project? Doctor came and educated you on BPSD?

• How would you describe your collaboration with your GP before the intervention?

- (How do they understand "cooperation"?)
- What was difficult/easy about the collaboration?

[UNDERSTANDING/COHERENCE]

• Has [name of nursing home doctor] taught you (and your colleagues) how to manage neuropsychiatric symptoms in residents with dementia?

• How did it work?

• What did you get out of the lesson?

[ENGAGEMENT/COGNITIVE PARTICIPATION]

Ongoing contact with nursing home GP

• Did you talk to colleagues about reducing psychotropic drugs for the citizens, who were involved in the project?

• Did you do anything to engage the rest of the staff to get involved?

• How did you keep the engagement going? Was a procedure put in place? Reminders?

Obstacles?
• Did something not work?

• Try to put into words the elements that have been an obstacle.

[COLLECTIVE ACTION]

This part is about how useful the material has been. We will talk about teaching at the care homes, preparation for the visits and the dialogue tool.

Nursing home teaching (of symptoms)

• What did you get out of the teaching session?

• Was more or less teaching needed?

Pre-visit reflection tool

Did you receive this form?

• How was it put into work?

• Where was it placed?

• How did the well-being scheme work?

• What was used/not used?

• Did you do anything before GP visitations?

• What did you get out of the reflection?

[ASSESSMENT/REFLEXIVE MONITORING]

• Compared to before the intervention - how has your collaboration with your GP changed?

We assume that you are also interested in reducing medication.

• From your chair, what would it take for less psychotropic medication to be prescribed?

• Why doesn't it happen?

• Have you experienced it happen? Elaborate.

• Where do the nursing home and the relatives fit into this? What is required of the collaboration?

• Do you have anything to add about your experience of the intervention here in conclusion that we have not covered?
