# Supplementary figures and images for: Factors promoting and impeding efforts to deprescribe antidepressants among nursing home residents with dementia– a process evaluation guided by normalization process theory
Source: BMC Nurs. 2024 Apr 28;23:287. doi: 10.1186/s12912-024-01932-x (PMC11057106; doi:10.1186/s12912-024-01932-x)

## Additional material 3: Coding Tree

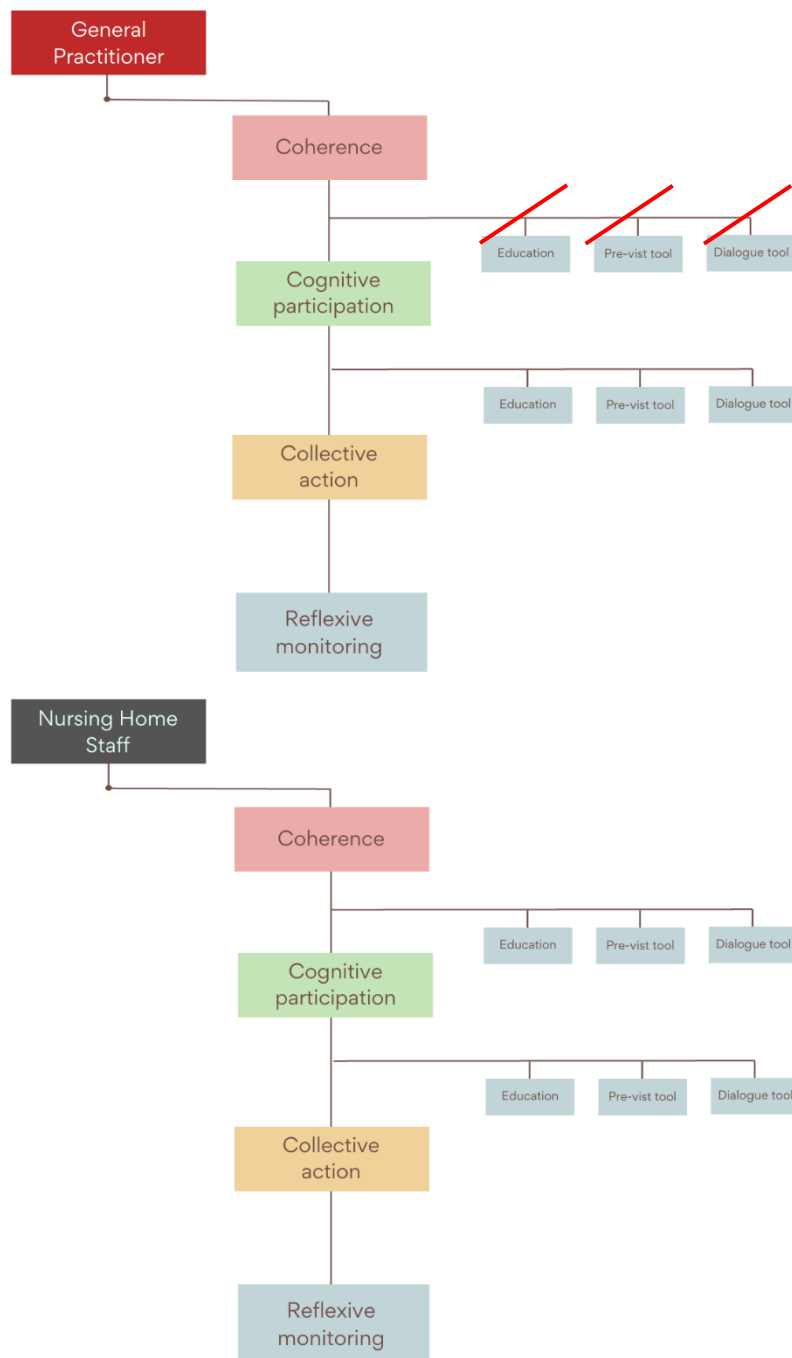

Supplement: Supplementary file 2 — Supplementary Material 2 [file 12912_2024_1932_MOESM2_ESM.pdf]
